# Screening and Selection of Medium Components for Cyclodextrin Glucanotransferase Production by New Alkaliphile* Microbacterium terrae* KNR 9 Using Plackett-Burman Design

**DOI:** 10.1155/2016/3584807

**Published:** 2016-02-03

**Authors:** Kiransinh N. Rajput, Kamlesh C. Patel, Ujjval B. Trivedi

**Affiliations:** ^1^Department of Microbiology and Biotechnology, School of Sciences, Gujarat University, Navrangpura, Ahmedabad, Gujarat 380 009, India; ^2^Department of Microbiology, BRD School of Biosciences, Sardar Patel Maidan, Sardar Patel University, Satellite Campus, Bakrol, Vallabh Vidyanagar, Gujarat 388 120, India

## Abstract

Cyclodextrin glucanotransferase (CGTase, EC 2.4.1.19) production using new alkaliphile* Microbacterium terrae* KNR 9 was investigated by submerged fermentation. Statistical screening for components belonging to different categories, namely, soluble and raw starches as carbon sources, complex organic and inorganic nitrogen sources, minerals, a buffering agent, and a surfactant, has been carried out for CGTase production using Plackett-Burman factorial design. To screen out *k* (19), number of variables, *k* + 1 (20), number of experiments, were performed. Among the fourteen components screened, four components, namely, soluble starch, corn flour, yeast extract, and K_2_HPO_4_, were identified as significant with reference to their concentration effect and corresponding *p* value. Although soluble starch showed highest significance, comparable significance was also observed with corn flour and hence it was selected as a sole carbon source along with yeast extract and K_2_HPO_4_ for further media optimization studies. Using screened components, CGTase production was increased to 45% and 87% at shake flask level and laboratory scale fermenter, respectively, as compared to basal media.

## 1. Introduction

Starch is an important storage compound synthesized by many plants as their carbon and energy source. Many bacteria can utilize starch as their carbon and energy reserve source. Cyclodextrins (CDs) are produced as a result of intramolecular transglycosylation (cyclization) reaction during degradation of starch by cyclodextrin glucanotransferase (CGTase, EC 2.4.1.19) enzyme. Cyclodextrins are cyclic oligosaccharides commonly composed of six, seven, or eight D-glucose units (*α*-, *β*-, and *γ*-cyclodextrins, resp.) joined by *α* 1,4-glycosidic bonds [1]. The CGTase is a multifunctional enzyme, catalyzing four different reactions: cyclization, disproportionation (cleavage of a linear maltooligosaccharide and transfer to a linear acceptor oligosaccharide), coupling (opening of the rings of CDs and transfer to a linear acceptor oligosaccharides), and weak hydrolysis reaction (production of a linear maltodextrin) [2].

A torus shaped microring of cyclodextrin molecule is having hydrophilic hydroxyl (-OH) groups on the outside of the ring molecule with the hydrophobic -CH groups and glycosidic oxygen located inside the cavity of the molecule. Because of this unique property, CDs can form molecular inclusion complexes with range of compounds and hence have found various applications [1, 3]. As a result of molecular complexation phenomenon, CDs can alter the solubility of a guest compound, stabilize against the effect of light, heat, and oxidation, mask unwanted physiological effects, and reduce volatility. CDs are used in many industrial productions, analytical methods, pharmaceuticals, food and flavors, cosmetics, packing, textiles, separation processes, and so forth [3–6]. *β*-Cyclodextrin and its derivatives are the most widely used form of cyclodextrins as it is commercially available and inclusion complexes can be prepared easily with *β*-CD [6].

CGTase is an extracellular enzyme, predominantly produced by strains of* Bacillus,* namely,* B*.* circulans* 251 [2],* B*.* macerans* [7],* B*.* firmus* [8, 9],* B*.* cereus* [10],* Bacillus stearothermophilus* HR1 [11], and* Bacillus* sp. TS1 [12]. Other known CGTase producing species are* Klebsiella pneumoniae* AS-22 [13],* Paenibacillus* sp. F-8 [14], and* Brevibacterium* sp. 9605 [15]. Thermostable CGTase is also reported from* Thermoanaerobacterium thermosulfurigenes* EM1 [16] and* Thermoanaerobacter* [17]. Georganta et al. [18] have reported the psychrophilic alkaliphile* Bacillus* sp. isolated from a deep sea mud sample for CGTase production. Majority of the CGTases produced by bacteria yield *β*-cyclodextrin as their major product in a mixture of other CDs in different ratios. Few CGTases have been reported to produce *α*- and *γ*-cyclodextrins as their major product along with other CDs [13, 15, 19].

Production of microbial metabolites can be increased by manipulating nutritional requirements, physical parameters, and genetic make-up of the producing strain [20]. Selection and incorporation of appropriate carbon, nitrogen, and other nutrient sources play an important role in designing the cost-effective production medium. Screening of various nutritional components can be done by two approaches, classical and statistical. In classical one-factor-at-a-time method, one independent variable is changed at a time keeping the others at fixed concentration or level. This approach is laborious and time consuming and, especially for a large number of variables, it does not ensure desirable conditions.

In contrast to this, statistical methods have several merits. They are rapid and reliable for short listings of nutrients, help in understanding the interactions among the nutrients at varying concentrations, and reduce the total number of experiments, resulting in saving of time, glassware, chemicals, and manpower [21]. For optimization of process conditions, many statistical factorial designs are available ranging from two-level factorial to multilevel factorial designs. If the number of ingredients to be screened is large during a fermentation process, it is complicated to screen them out by multifactorial designs as it offers large number of experiments (2^*k*^, where *k* is the total number of variables). The Plackett-Burman statistical method is a two-level factorial design used to study the effect of *k* variables in *k* + 1 experiments [22]. This method is used for initial screening of the ingredients and to understand their significance on the product formation and then significant ingredients are selected for further media optimization [20].

CGTase has been commonly produced by submerged fermentation in media containing various types of starch or other carbohydrates and complex nitrogen sources [23]. Some authors have reported the screening of various carbon and nitrogen sources by classical one-factor-at-a-time method followed by statistical optimization of media [13, 24, 25]. The aim of the present work was to screen out fourteen media components for their influence on CGTase production using a new alkaliphile isolate* Microbacterium terrae* KNR 9 using Plackett-Burman design.

## 2. Materials and Methods

### 2.1. Materials

*β*-Cyclodextrin was purchased from Himedia, Mumbai, India. Soluble starch, yeast extract, and peptone were obtained from Qualigens, India. Phenolphthalein was purchased from Merck India Ltd. Finely grounded corn flour and rice flour were collected from local flour mills and were sieved through fine cheese cloth to get fine uniform raw starch powder. All other chemicals were of analytical grade.

### 2.2. Organism

The CGTase producing alkaliphilic bacterial culture used in this study was isolated from soil as described by Park et al. [26]. This natural bacterial isolate was identified as* Microbacterium terrae* KNR 9 by IMTECH, Chandigarh, India (deposited at the same institute as* Microbacterium terrae* MTCC 8083). It was grown on the basal media containing (g/L) soluble starch, 20; peptone, 5; yeast extract, 5; K_2_HPO_4_, 1; MgSO_4_·7H_2_O, 0.2; agar, 20; and Na_2_CO_3_, 20 (autoclaved separately) at 30°C for 24 h and maintained at 4°C by periodic transfer.

### 2.3. Inoculum Preparation

The culture was transferred in a 250 mL flask containing 50 mL inoculum medium (same as above except agar) from a slant culture and incubated at 30°C, 150 rpm for 24 h. Cells were then harvested in sterile centrifuge tubes (15 mL) at 5000 rpm for 10 min. Pellet of cells was resuspended in sterile medium to get an optical density (OD) of 1.0 at 660 nm and used as an inoculum. A 3.0% (v/v) inoculum was transferred to flask containing production medium under aseptic condition.

### 2.4. Production Media

Fourteen components of different categories were included in the production medium at two levels (Table 1). 100 mL production media with different nutrient combinations were prepared in 250 mL flasks as shown in the experimental design (Table 2) and sterilized at 121°C for 15 minutes. All the components were dissolved in distilled water except for corn flour, rice flour, and soybean meal which were weighed and directly added to the media. The pH of media was adjusted to alkaline range (>10.0) by adding separately sterilized 2.0% (w/v) Na_2_CO_3_ in all the media flasks. Inoculated flasks were incubated at 30°C and 150 rpm on a rotary shaker. Media flasks were harvested after 72 h, cells and suspended particles were removed by centrifugation, and the supernatant was used to determine CGTase activity.

### 2.5. CGTase Assay

CGTase activity was determined by phenolphthalein assay method described by Goel and Nene [8] with minor modification. 100 *μ*L approximately diluted enzyme was incubated with 1.0 mL of 50 mg soluble starch in sodium phosphate buffer (pH 6.0, 0.05 M) at 60°C for 30 min. The reaction was stopped by quickly cooling the tubes on ice. Four milliliters of working phenolphthalein solution was added, the tubes were vortexed, and the absorbance of the mixture was immediately measured at 550 nm. The working phenolphthalein solution was made by adding 1 mL of phenolphthalein stock (4 mM in ethanol) to 100 mL of 125 mM Na_2_CO_3_ prepared in 4% ethanol. The standard *β*-cyclodextrin estimation was also carried using the same method. One unit was defined as the amount of enzyme that produced one *μ*mole of *β*-cyclodextrin per min.

### 2.6. Statistical Experimental Design and Analysis of the Data

Plackett-Burman design was used to evaluate the relative importance of various media components including soluble and raw starches, organic and inorganic nitrogen sources, a phosphorous source/buffering agent, minerals, and a surfactant for CGTase production in submerged fermentation. This design was chosen to screen out the important media components with respect to their main effects and not the interaction effects.

In Plackett-Burman design, the total number of experiments to be carried out is *k* + 1, where *k* is number of variables (media components). For total of nineteen variables (*k* = 19) including fourteen media components (assigned variables) (Table 1) and five dummy variables (unassigned variables), twenty-run design was generated (Table 2). The incorporation of dummy variables in an experiment makes it possible to estimate the variance of an effect (experimental error). Normally three dummy variables will provide an adequate estimate of the error. However, more dummy variables can be used if fewer real variables need to be studied in an investigation [27]. In the design of experiments each horizontal raw represents a trial run of experimental production media with different combination of nutritional components and each vertical column represents an independent (assigned) or dummy (unassigned) variable. All the fourteen components were represented at two levels, higher concentration (+) and lower concentration (−), in the production media. Care must be taken during setting the difference between high and low level, as a small differential may not show any effect and a large differential for a sensitive component can mask other components [28]. In the present study, the components of basal media, that is, soluble starch, peptone, yeast extract, K_2_HPO_4_, and MgSO_4_·7H_2_O, were added at both factorial levels, lower and higher concentration, whereas the remaining other components were added at their higher level only; lower level of them was adjusted to zero. The concentration effect of each variable was determined by the following equation:

(1) $$\begin{array}{c} E\left(\;Xi\;\right)=\frac{2\left(\Sigma {M_{i}}^{+}-\Sigma {M_{i}}^{-}\right)}{N}, \end{array}$$

where *E*(*Xi*) is concentration effect of the variable, *M*_*i*_^+^ and *M*_*i*_^−^ are the CGTase production of the trials, where the variable (*Xi*) estimated is present at its higher and lower concentrations, respectively, and *N* is the number of trials (20). Experimental error was estimated by calculating the variance of dummy variables as follows:

(2) $$\begin{array}{c} \text{Veff}=\frac{\Sigma {\left(ED\right)}^{2}}{n}, \end{array}$$

where Veff is the variance of the concentration effect (experimental error), *ED* is the concentration effect of the dummy variable, and *n* is the number of dummy variables. The standard error (SE) of the concentration effect was the square root of the variance of dummy variables and the significance level (*p* value) of each concentration effect was determined using Student's *t*-test:

(3) $$\begin{array}{c} t\left(\;Xi\;\right)=\frac{E\left(Xi\right)}{\text{SE}}, \end{array}$$

where *E*(*Xi*) is the effect of variable *Xi*.

Statistical confidence level of each variable was calculated as

(4) $$\begin{array}{c} \text{Statistical confidence}=\left(\;1-p\;\right)\times 100. \end{array}$$

A value of *p* = 0.05 corresponds to a statistical confidence level of 95% and hence any component showing a statistical confidence level higher than 95% was considered as significant.

## 3. Result and Discussion

To formulate economic CGTase production media, fourteen components of different categories were screened using the Plackett-Burman design. Among the carbon sources, soluble starch and raw organic carbon sources like corn flour and rice flour were selected for the study. Organic and inorganic nitrogen sources like peptone, yeast extract, soybean meal, casein hydrolysate, ammonium sulphate, and ammonium nitrate were added in the media to test their significance. K_2_HPO_4_ was added as a buffering agent. Three minerals MgSO_4_·7H_2_O, CaCl_2_·2H_2_O, and FeSO_4_·7H_2_O and a surfactant Tween-80 were included in the study as medium components.

CGTase production in 20 experimental runs at 30°C, 150 rpm, after 72 h is represented in Table 2. Experimental analysis was carried out by calculating the concentration effect (*E*), standard error (SE), *t*-value, *p* value, and statistical confidence level (%) of each component and is represented in Table 3. Although screening of significant components was done on the basis of their positive concentration effect showing more than 95% confidence, the positive or negative concentration effect of a variable is equally important and helpful in deciding further increase or decrease in the concentration of a particular variable in subsequent media optimization studies. If the concentration effect of a tested variable is positive, the influence of the variable on the production is greater at its higher concentration and if it is negative, the influence of the variable is greater at its lower concentration.

As revealed from Table 3, soluble starch, corn flour, rice flour, yeast extract, soybean meal, casein hydrolysate, K_2_HPO_4_, MgSO_4_·7H_2_O, CaCl_2_·2H_2_O, FeSO_4_·7H_2_O, and Tween-80 are having positive concentration effect on CGTase production, whereas peptone, (NH_4_)_2_SO_4_, and (NH_4_)NO_3_ are having negative concentration effect. Among the components showing positive concentration effect soluble starch, corn flour, yeast extract, and K_2_HPO_4_ have shown confidence level of more than 95% and hence were considered as significant. Rest of the components, namely, rice flour, soybean meal, casein hydrolysate, MgSO_4_·7H_2_O, CaCl_2_·2H_2_O, FeSO_4_·7H_2_O, and Tween-80, were insignificant, having confidence level below 95%.

Starch is the most important carbon source commonly used for the CGTase production. Goel and Nene [8] have reported the CGTase production by* B*.* firmus* during raw tapioca starch degradation. Significance of sago starch as carbon source has been reported using* B*.* stearothermophilus* HR1 [11] and* Bacillus* sp. TSl-1 [12]. Optimization of medium for CGTase production using tapioca starch was reported by Ibrahim et al. [25]. However, CGTase production using sugars has been reported, where xylose showed the maximum enzyme production [10]. In our study, an attempt was made to compare the influence of corn flour and rice flour with soluble starch for CGTase production. Soluble starch, rice flour, and corn flour have shown the significance of 98.06%, 94.68%, and 97.48% confidence level, respectively.* Mic. terrae* KNR 9 was able to utilize various starches and among them corn flour has shown the confidence level of 97.48% which is as good as soluble starch (98.06%). Therefore, soluble starch and corn flour both were included as a sole carbon source in further media optimization studies.

Among the complex organic nitrogen sources tested, yeast extract was found to be most significant component contributing to the synthesis of CGTase at a confidence level of 98.82%. Gawande and Patkar [13] have reported the importance of yeast extract in media for CGTase production by* Klebsiella pneumoniae* AS-22. Yeast extract showed an appreciable effect on CGTase production, presumably due to the presence of some essential nutrients or inducers. Soybean meal and casein hydrolysate showed the positive effect but were found insignificant below 95% confidence level. Peptone was the only organic nitrogen source showing the negative concentration effect. Inorganic nitrogen sources (NH_4_)_2_SO_4_ and (NH_4_)NO_3_ were having very high negative concentration effect and can be excluded during further studies. Interestingly no growth of* Mic. terrae* KNR-9 was observed when inorganic nitrogen salts were supplied as a sole nitrogen source (data not shown). Thatai et al. have reported the repression of CGTase production in presence of ammonium salts [29]. However there is a report for the production of CGTase using various ammonium salts by* B*.* circulans* DF 9R [24].

K_2_HPO_4_ was found significant at 97.87% confidence level, as it is a good buffering agent as well as phosphorus source. Ibrahim et al. have reported the importance of K_2_HPO_4_ in the media for CGTase production by* Bacillus* sp. G1 [25].

Confidence levels of minerals like MgSO_4_·7H_2_O, CaCl_2_·2H_2_O, and FeSO_4_·7H_2_O were below 95% and hence were considered as insignificant. MgSO_4_·7H_2_O has been reported as an essential mineral element for CGTase production [25]. Importance of magnesium as an essential element for bacterial growth and iron for CGTase production has been demonstrated using* B*.* circulans* DF 9R [24]. In our screening study, mineral requirements for CGTase production might have been fulfilled by natural organic carbon and nitrogen sources; hence external supply of these minerals had no significant effect. Meat and plant infusions are aqueous extracts that are commonly used as sources of nutrients for the cultivation of microorganisms and they contain amino acids and lower molecular weight peptides, carbohydrates, vitamins, minerals, and trace elements [30]. A surfactant Tween-80 was also found insignificant as it has confidence level below 95%. However, it has been reported to increase the enzyme production [31].

**Figure 1:**
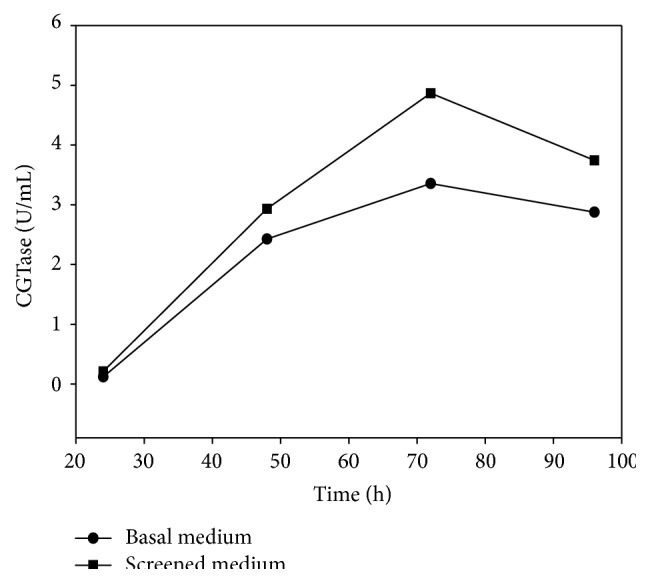
Comparative CGTase production in basal medium and screened medium at shake flask level.

**Figure 2:**
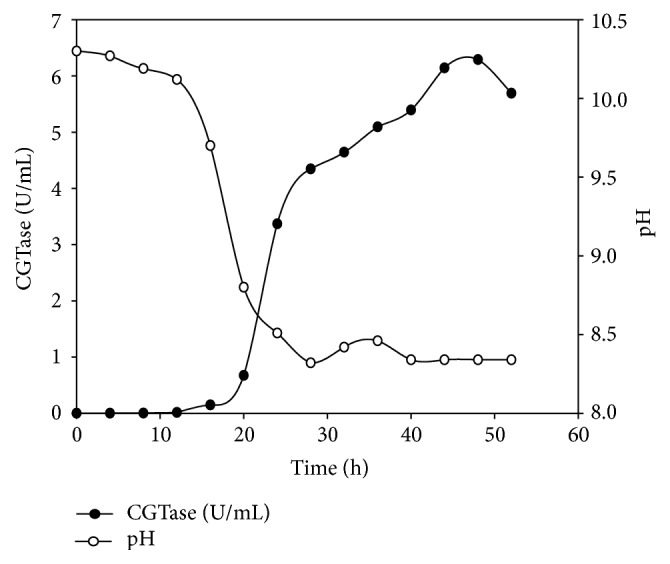
CGTase production in laboratory scale fermenter.

Comparative CGTase production has been carried out with basal medium as well as medium containing screened components (g/L): corn flour, 20; yeast extract, 10; K_2_HPO_4_, 1; and Na_2_CO_3_, 20 (autoclaved separately) at shake flask level (50 mL medium in a 250 mL flask) and laboratory scale fermenter (2.5 lit medium in a 5 lit fermenter vessel). At shake flask level, maximum CGTase production of 3.36 U/mL and 4.87 U/mL was obtained at 30°C, 150 rpm, after 72 h with the basal medium and screened components confirming medium, respectively (Figure 1). In the fermenter, maximum CGTase production of 6.29 U/mL was achieved at 30°C after 48 h of incubation (Figure 2). Laboratory scale fermenter showed increased CGTase production with reduction in incubation time. Using the screened media components, CGTase production has been increased to about 45% at shake flask level and 87% at laboratory scale fermenter, respectively, as compared to basal medium.

Use of Plackett-Burman design has been reported for screening of media components for production of lactic acid [32, 33], alpha-galactosidase [21], and riboflavin by* Eremothecium ashbyii* [34]. Using the same design, increased yield of thermostable *β*-amylase and pullulanase has been reported with* Clostridium thermosulfurogenes* SV2 in solid state fermentation [35]. Apart from the objective of product enhancement, Plackett-Burman design has been successfully used for screening of media components to achieve the spore production of* Colletotrichum coccodes* [36] and desired growth rate for aggregated shipworm bacterium [37].

## 4. Conclusion

Thus the present study shows successful application of Plackett-Burman design for statistical screening of media components and selection of the significant media components for CGTase production by a newly isolated alkaliphile* Mic. terrae* KNR-9. Application of this design has not only helped in short listing and selecting the best variables such as soluble starch, corn flour, yeast extract, and K_2_HPO_4_ for CGTase production but also ensured the better CGTase production. The results of this study have scope for improvement in CGTase production by further optimization of the most significant components.

## Figures and Tables

**Table 1:** Variables showing medium components used in Plackett-Burman design.

Variables	Medium components	(−) Lower concentration (g/L)	(+) Higher concentration (g/L)
*X*1	Soluble starch	1.0	10.0
*X*2	Rice flour	0.0	10.0
*X*3	Corn flour	0.0	10.0
*X*4	Peptone	1.0	10.0
*X*5	Yeast extract	1.0	10.0
*X*6	Soybean meal	0.0	10.0
*X*7	Casein hydrolysate	0.0	10.0
*X*8	(NH_4_)_2_SO_4_	0.0	2.5
*X*9	(NH_4_)NO_3_	0.0	2.5
*X*10	K_2_HPO_4_	0.2	2.0
*X*11	MgSO_4_·7H_2_O	0.025	0.25
*X*12	CaCl_2_·2H_2_O	0.0	0.1
*X*13	FeSO4·7H_2_O	0.0	0.1
*X*14	Tween-80	0.0	100 (*μ*L)

**Table 2:** CGTase production in experimental runs of Plackett-Burman design, where *X*1 ⋯ *X*14 are independent variables and *D*1 ⋯ *D*5 are dummy variables.

Run	Components																			
	*X*1	*X*2	*X*3	*X*4	*X*5	*X*6	*X*7	*X*8	*X*9	*X*10	*X*11	*X*12	*X*13	*X*14	*D*1	*D*2	*D*3	*D*4	*D*5	CGTase (U/mL)
1	+	+	−	−	+	+	+	+	−	+	−	+	−	−	−	−	+	+	−	6.391
2	−	+	+	−	−	+	+	+	+	−	+	−	+	−	−	−	−	+	+	0.034
3	+	−	+	+	−	−	+	+	+	+	−	+	−	+	−	−	−	−	+	0.032
4	+	+	−	+	+	−	−	+	+	+	+	−	+	−	+	−	−	−	−	0.011
5	−	+	+	−	+	+	−	−	+	+	+	+	−	+	−	+	−	−	−	6.490
6	−	−	+	+	−	+	+	−	−	+	+	+	+	−	+	−	+	−	−	4.693
7	−	−	−	+	+	−	+	+	−	−	+	+	+	+	−	+	−	+	−	0.429
8	−	−	−	−	+	+	−	+	+	−	−	+	+	+	+	−	+	−	+	0.016
9	+	−	−	−	−	+	+	−	+	+	−	−	+	+	+	+	−	+	−	4.293
10	−	+	−	−	−	−	+	+	−	+	+	−	−	+	+	+	+	−	+	3.149
11	+	−	+	−	−	−	−	+	+	−	+	+	−	−	+	+	+	+	−	0.022
12	−	+	−	+	−	−	−	−	+	+	−	+	+	−	−	+	+	+	+	1.008
13	+	−	+	−	+	−	−	−	−	+	+	−	+	+	−	−	+	+	+	8.188
14	+	+	−	+	−	+	−	−	−	−	+	+	−	+	+	−	−	+	+	4.843
15	+	+	+	−	+	−	+	−	−	−	−	+	+	−	+	+	−	−	+	9.137
16	+	+	+	+	−	+	−	+	−	−	−	−	+	+	−	+	+	−	−	2.945
17	−	+	+	+	+	−	+	−	+	−	−	−	−	+	+	−	+	+	−	2.346
18	−	−	+	+	+	+	−	+	−	+	−	−	−	−	+	+	−	+	+	3.994
19	+	−	−	+	+	+	+	−	+	−	+	−	−	−	−	+	+	−	+	2.596
20	−	−	−	−	−	−	−	−	−	−	−	−	−	−	−	−	−	−	−	6.391

**Table 3:** Calculated concentration effect, standard error, *t*-value, *p* value, and confidence level.

Components	Concentration effect	Standard error	*t*-value	*p* value	Confidence (%)
Soluble starch	1.6299	0.4797	3.3971	0.0193	98.06
Rice flour	1.2091	0.4797	2.5200	0.0531	94.68
Corn flour	1.5145	0.4797	3.1566	0.0251	97.48
Peptone	−1.4823	0.4797	−3.0895	0.0272	97.28
Yeast extract	1.8579	0.4797	3.8723	0.0117	98.82
Soybean meal	1.1973	0.4797	2.4955	0.0547	94.52
Casein hydrolysate	0.5583	0.4797	1.1636	0.2970	70.29
Ammonium sulfate	−2.6571	0.4797	−5.5381	0.0026	99.73
Ammonium nitrate	−2.6921	0.4797	−5.6110	0.0025	99.75
K_2_HPO_4_	1.5881	0.4797	3.3100	0.0212	97.87
MgSO_4_·7H_2_O	0.0293	0.4797	0.0610	0.9536	4.63
CaCl_2_·2H_2_O	0.5505	0.4797	1.1473	0.3031	69.68
FeSO_4_·7H_2_O	0.0891	0.4797	0.1857	0.8599	14.00
Tween-80	0.4845	0.4797	1.0098	0.3589	64.10
